# On the Several Molecules and Nanostructures of Water

**DOI:** 10.3390/ijms13011066

**Published:** 2012-01-19

**Authors:** Cynthia Kolb Whitney

**Affiliations:** 141 Rhinecliff Street, Arlington, MA 02476, USA; E-Mail: Galilean_Electrodynamics@comcast.net; Tel.: +1-781-643-3155; Fax: +1-781-643-3155

**Keywords:** Algebraic Chemistry, Brown’s gas, boiling, chemical bond, cold fusion, energy storage, freezing, ice, ionization potentials, melting, quantum chemistry, poly water, quantum mechanics, snow flakes, water chemistry

## Abstract

This paper investigates the water molecule from a variety of viewpoints. Water can involve different isotopes of Hydrogen and Oxygen, it can form differently shaped isomer molecules, and, when frozen, it occupies space differently than most other substances do. The tool for conducting the investigation of all this is called ‘Algebraic Chemistry’. This tool is a quantitative model for predicting the energy budget for all sorts of changes between different ionization states of atoms that are involved in chemical reactions and in changes of physical state. The model is based on consistent patterns seen in empirical data about ionization potentials, together with rational scaling laws that can interpolate and extrapolate for situations where no data are available. The results of the investigation of the water molecule include comments, both positive and negative, about technologies involving heavy water, poly water, Brown’s gas, and cold fusion.

## 1. Introduction

Human beings are made mostly of water, and that perspective makes water a subject fitting for deep mathematical study. By ‘deep’ I mean looking at many possible ionic structures, and investigating the possible macroscopic consequences thereof. Water does a number of interesting things, not all of which we are intimately familiar with, since we live mostly within the circumstances of planet Earth’s surface. But we do know about interesting behaviors of some other molecules that are, in deep ways, similar to water. So this study includes some of those other molecules, to ponder as analogs of water, or *vice versa*, and guide us to look for behaviors we already know about from the other molecules. So this study includes, not just water, but also methane, other hydrocarbons, and other atoms/molecules involved with water in various technologies.

The analytical tool used throughout is ‘Algebraic Chemistry’ (AC). This is a quantitative model for predicting the energy budget for all sorts of changes between different ionization states of atoms that are involved in molecules or nanostructures undergoing chemical reactions or changes of physical state. This paper uses the model to develop information about water: its several possible isomer molecules, the ways in which they might interact, their implications in regard to macroscopic physical states. Analogies between water and other simple molecules are highlighted.

Section 2 reviews and expands information of a formulaic nature from [1], and, following the style of [2], introduces data in numerical form (included as Appendix 1). Section 3 applies AC to ordinary water in its liquid state. Section 4 discusses water in its different physical states, including solid state, which has to initiate with a nanostructure. Section 5 applies AC to discuss water molecules in unusual isomers, arguing that these isomers arise from unusual electron configurations. This leads to a discussion of the phenomenon known as ‘Brown’s Gas’ [3,4], and some parallels with hydrocarbons [5,6]. Section 6 discusses water molecules formed with isotopes of its constituent atoms. This leads to a discussion of the phenomenon known as ‘Cold Fusion’ [7,8]. Section 7 summarizes conclusions, and Appendix 1 collects numerical data in a convenient form for future use by readers.

## 2. Algebraic Chemistry

All atoms possess ‘Ionization Potentials’ that reflect the strength with which electrons are bound to them. Ionization potentials are generally measurable, and data about them is fairly abundant. As shown in [1], ionization potentials fall into reliable patterns that can be characterized algebraically. Ionization potentials constitute the basic information needed to support the calculations in the following Sections. So we review the formulaic information here, and present it in numerical form, convenient for current and future calculations, in Appendix 1. Some of the formulaic information is from [1], but some is new. The information that is newly developed since [1] is:

- **Higher-Order Ionization Potentials: These are rewritten to highlight use of ‘population-generic’ information and ‘element-specific’ information.**
- **Ionization Potentials of Ions: These are developed here, starting from ‘population-generic information and ‘element-specific’ information.**

### 2.1. Patterns in Ionization Potentials

Data on ionization potentials for most elements are generally available for at least several ionization orders. It has been discovered that, after applying a simple scaling, the data fall into a neat pattern. The scaling applied to a measured ionization potential is *M/Z*, where *M* is nuclear mass, and *Z* is nuclear charge. The resulting number is represented by the symbol *IP_IO,Z_*, where integer *IO* is the ionization order. Like the raw data from which it arises, it is denominated in electron volts.

**Figure 1:**
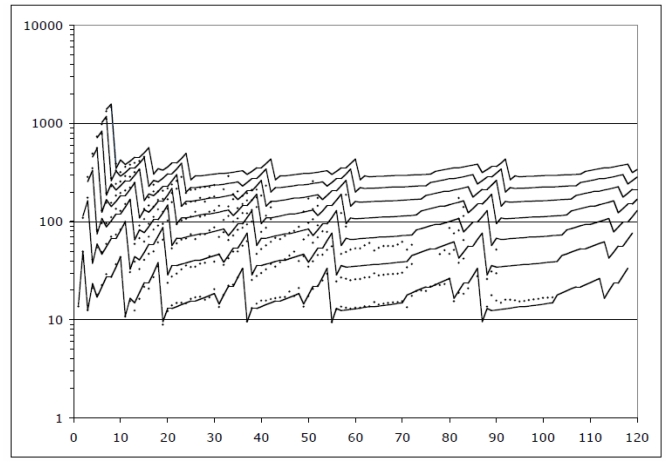
Ionization potentials, scaled appropriately and modeled algebraically.

Figure 1 (Fig. 2 in [1]) depicts the behavior of *IP*’s for all elements (nuclear charge *Z* = 1 to *Z* = 120 shown). Element *Z* actually allows *Z* ionization potentials, but for larger *Z*, many *IP*’s are not so easy to measure. Readily available data go only to seventh order, so that is how many orders are shown. The model is capable of producing plausible estimates for all *M/Z* -scaled *IP*’s for all *IO*’s, beyond those measured, and all *Z*’s, beyond those yet demonstrated to exist.

The points on Figure 1 are the *IP* data points. The lines on Figure 1 represent the algebraic model for the *IP*’s. The model consists of straight-line segments on a semi log plot. That implies simple power laws.

The most striking fact revealed by Figure 1 is that, apart from its first point, the curve for *any IO* is quite like the curve for *IO* = 1, but shifted right by *IO* − 1 elements, and muted in amplitude, more and more muted as *IO* increases.

The rightward shift of each model line means that each successive element reveals one additional bit of information about *all* subsequent elements. This fact speaks to the universality of chemical information: basic information about any one element can be inferred reliably from basic information about other elements.

The amplitude muting of successive model lines means the higher-order *IP*’s involve functions of *IO* that are not just linear, or just quadratic, or any one power. This fact speaks to the subtlety of chemical information!

### 2.2. First-Order Ionization Potentials

Since first-order ionization potentials determine everything else, let us begin with ionization order *IO* = 1. The most startling fact uncovered is that the total rise on *every* period is the same::

(1) Total rise on every period=7/2

The second most startling fact is that the fall from one period to the next is apparently very regular:

(2) Period to period drop=7/8 three times;then 1 three times

On each period rise, there are sub-period rises. They are all of the form

(3) Incremental rise=total rise×fraction

For the first sub-periods,

(4) Fraction=1,1/2,1/3;then 1/4 four times

For all subsequent sub-periods,

(5) $$\text{fraction}=[(2l+1)/N^{2}][(N-l)/l]$$

where *l* is the traditional ‘angular momentum’ quantum number for nominal single-electron states being filled, and *N* is a non-traditional parameter that depends on the number *L* of elements in a period: 2*N*^2^ = *L*. Table 1 collects all the information about sub-period fractional rises.

In the model, sub-period rises are split between the two spin states, plus 1/2 and minus 1/2. That makes two distinct sub-segments. Further details and actual numerical data are given in Appendix 1.

### 2.3. Higher-Order Ionization Potentials

The model for atoms used here is based on Hydrogen. The Hydrogen-based model invites the division of each ionization potential *IP*_1_*_,Z_* into two parts, one being *IP*_1,1_ for the generic Hydrogen-like collective interaction with the nucleus, and the other being the increment Δ*IP*_1_*_,Z_* = *IP*_1_*_,Z_* − *IP*_1,1_ for the element-specific electron-electron interactions, which do not exist for Hydrogen.

**Figure 2:**
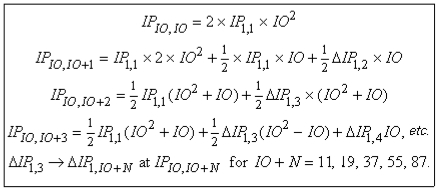
Behavior of higher-order *IP*’s (*IO* > 1).

Higher ionization order is characterized by *IO* > 1. Figure 2 uses *IP*_1,1_ and the *IP*_1_*_,Z_* to express the *IP*’s for all *IO* > 1. (Ref. [1] gave a detailed development of the *IP*’s for *IO* > 1 and presented the information without using the decomposition of *IP*_1_*_,Z_* into *IP*_1,1_ and *IP*_1_*_,Z_*.)

The ‘*etc*.’ means that the pattern established at *IP_IO,IO_*_+3_ continues from there on. Indeed, even *IP_IO,IO_*_+2_ is a special case of the general pattern revealed in *IP_IO,IO_*_+3_. The first term in *IP_IO,IO_*_+3_ is universal. The second term is period specific. The third term is element specific.

The leading terms representing any *IP* are proportional to *IO*^2^. This dependence suggests that the physical process generating the data involves removing *IO* electrons all at once, and not removing just the single electron left after *IO* − 1 other electrons have already been removed, or possibly just skipped over and left in place. This distinction about removing *IO* electrons all at once, *vs*. any scenario that removes just one electron, is obvious from the mathematical factor *IO*^2^, but it is not obvious from a description by a typical text phrase, such as ‘third-order ionization potential’, for example. So when reading the existing literature on ionization potentials, always watch out for the possibility of confusion arising from inadequate language.

The secondary terms representing any arbitrary *IP* are linear in *IO*. These terms implement the ‘shift right’ behavior seen in Figure 1. They also determine the numerical pattern that the period rises follow. For *IO* ≡ 1, the period rises are *all* 7/2. For all *IO >* 1, all but the first period are the same, but less than 7/2, gradually approaching unity as *IO* increases.

A formula for the period rises can be determined by looking at the ratio *IP*_*IO*,*IO*+10_/*IP*_*IO*,*IO*+2_:

(6) $$\frac{{IP}_{IO,IO+10}}{{IP}_{IO,IO+2}}=\frac{\frac{1}{2}{IP}_{1,1}({IO}^{2}+IO)+\frac{1}{2}\Delta {IP}_{1,3}({IO}^{2}-IO)+\Delta {IP}_{1,10}IO}{\frac{1}{2}{IP}_{1,1}({IO}^{2}+IO)+\frac{1}{2}\Delta {IP}_{1,3}\times ({IO}^{2}+IO)},$$

Here $\Delta {IP}_{1,3}=-\frac{1}{8}{IP}_{11}$ and $\Delta {IP}_{1,10}=\left(\frac{7}{8}\times \frac{7}{2}-1\right){IP}_{1,1}=\frac{33}{16}{IP}_{1,1}$. From these values, one finds that period rises, except for the first period, follow the simple formula (*IO +* 6)/(*IO +* 1), which reduces to 7/2 for *IO* = 1.

The exception for first-period rises when *IO >* 1 demands an explanation. To begin developing the explanation, note the factor of 2 in the formula for *IP_IO,IO_* for *IO >* 1:

(7) $${IP}_{IO,IO}=2\times {IP}_{1,1}\times {IO}^{2}$$

Why does this formula not come out, as it must for *IO* ≡ 1, as *IP_IO_*_,_*_IO_* ≡ *IP*_1,1_ × *IO*^2^ ? A possible explanation is that, unlike first-order ionizations, these *total* ionizations are *not* best characterized as ‘removal of electrons from the atomic system’; they are *better* characterized as ‘expulsion of the *nucleus* from the atomic system’. Note that electrons have a lot of kinetic energy; on average, an amount equal to half the magnitude of their (negative) potential energy in the atomic system. By contrast, the nucleus has almost *no* kinetic energy. So expelling the nucleus from the atomic system takes essentially *twice* the energy that removing all of the electrons *together* from the atomic system would take. But the fragility of the electron subsystem probably prevents the latter scenario.

Next, consider the formula for the scenario that starts with *IO +* 1 electrons, and ends up with one electron:

(8) $${IP}_{IO,IO+1}={IP}_{1,1}\times 2\times {IO}^{2}+\frac{1}{2}\times {IP}_{1,1}\times IO+\frac{1}{2}\Delta {IP}_{1,2}\times IO$$

The first term, *IP*_1,1_ × 2 *× IO*^2^, is identical to the formula for starting with *io* electrons and ending up with no electrons. So this part of the scenario looks like the blow-the-nucleus-out scenario, but not with just the nucleus alone; instead, this nucleus takes one electron with it, so that what is blown out is a nucleus-plus-electron system that is like a nucleus with charge *IO* instead of *IO* + 1. That leaves an electron system with *IO* electrons still to dispose of.

The second term, $\frac{1}{2}\times {IP}_{1,1}\times IO$, defines what it means to ‘dispose of’ *IO* electrons. Each electron is blown away with enough energy, $\frac{1}{2}\times {IP}_{1,1}$, so that any pair of them has energy *IP*_1,1_, sufficient to keep them away from each other.

The third term, $\frac{1}{2}\Delta {IP}_{1,2}\times IO$, suggests a possible structure for the system of *IO* electrons. If the system is a ring, or several rings, it takes *IO* binary cleavages to destroy the system. So there is the factor of *IO* multiplying Δ*IP*_1_*_,_*_2_. But why is there also the factor of $\frac{1}{2}$ multiplying Δ*IP*_1_*_,_*_2_? Well, consider that when every electron gets energy $\frac{1}{2}\Delta {IP}_{1,2}$, then any pair of them has enough energy, Δ*IP*_1_*_,_*_2_, to resist reuniting as a pair.

Next, consider the scenario that starts with *IO* + 2 electrons, and ends up with 2 electrons. We have:

(9) $${IP}_{IO,IO+2}=\frac{1}{2}{IP}_{1,1}({IO}^{2}+IO)+\frac{1}{2}\Delta {IP}_{1,3}\times ({IO}^{2}+IO)$$

The first term, $\frac{1}{2}{IP}_{1,1}({IO}^{2}+IO)$, contains $\frac{1}{2}{IP}_{1,1}{IO}^{2}$, which is quite different from the *IP*_1,1_ × 2 *× IO*^2^ seen in the cases of *IO* and *IO* + 1: it has the overall factor of $\frac{1}{2}$ instead of 2. This means that there is no expulsion of a nucleus-like system consisting of the nucleus plus two electrons, which would have charge *IO* instead of *IO* + 2. Instead, *IO* electrons are blown away from the atom, leaving the nucleus-like system behind. The factor of $\frac{1}{2}$ on $\frac{1}{2}{IP}_{1,1}{IO}^{2}$ means the nucleus-like system and the expelled electron system have between them enough energy, *IP*_1,1_*IO*^2^, to keep them away from each other.

The first term here also contains $\frac{1}{2}{IP}_{1,1}\times IO$, which was present in the *IO* + 1 case as the second term there. The $\frac{1}{2}{IP}_{1,1}\times IO$ means the same thing here: each electron is given enough energy, $\frac{1}{2}\times {IP}_{1,1}$, so that any pair of them has energy *IP*^1,1^, sufficient to keep them away from each other.

The second term here, $\frac{1}{2}\Delta {IP}_{1,3}\times ({IO}^{2}+IO)$, is actually negative, because $\Delta {IP}_{1,3}=-\frac{1}{8}{IP}_{11}$. The second term scales down the energy increments recommended by the first term as being necessary to keep away the *IO* electrons, either as a group (the *IO*^2^ term), or as individuals (the *IO* term). The scale-down reflects the fact that an electron system consisting of just two electrons by themselves is the most stable electron system seen anywhere in Nature. Like a paper currency note, it can seem too big to break.

Next, consider the scenario that starts with *IO* + 3 electrons and ends up with 3 electrons. We have:

(10) $${IP}_{IO,IO+3}=\frac{1}{2}{IP}_{1,1}({IO}^{2}+IO)+\frac{1}{2}\Delta {IP}_{1,3}({IO}^{2}-IO)+\Delta {IP}_{1,4}IO$$

The first term is the same as it was in *IP_IO,IO_*_+2_, and has the same meaning here. The second term, $\frac{1}{2}\Delta {IP}_{1,3}({IO}^{2}-IO)$, differs by the internal minus sign from the second term $\frac{1}{2}\Delta {IP}_{1,3}({IO}^{2}+IO)$ in *IP_IO,IO_*_+2_. That minus sign on the already negative Δ*IP*_1,3_ effectively adds to the energy requirement to keep individual electrons from coming back. The third term, Δ*IP*_1,4_*IO*, further raises the energy needed to keep individual electrons from coming back, inasmuch as Δ*IP*_1,4_ is positive. The message is: three electrons is a really an unstable situation. As with small coins, something is likely to get lost.

### 2.4. Ionization Potentials of Ions

The events described by higher-order ionization potentials are very vigorous, even violent: stripping *IO* electrons off an atom all at once. This takes explosions, particle beams, *etc*. There is a much gentler way to end up with the same end result: strip the electrons off one at a time. (Or add them one at a time.) The distinction is usually not made clear, but it is well captured in [2] with the attribution ‘Pauling’ for the ‘all-at-once’ event, and the word ‘spectroscopic’ for the ‘one-at-a-time’ process.

The gentle subtractions or additions are what most chemical reactions do. So we need to work out how to model them. Above we separated Δ*IP*_1,1_ and Δ*IP*_1,_*_Z_*. For first-order ionizations, the *IP*’s scale with *Z/M*. This means that the constituent parts, *IP*_1,1_ and Δ*IP*_1,_*_Z_*, both scale with *Z/M* too. But for an already ionized atom, the modifications to *Z/M* will be different for the two parts, *IP*_1,1_ and Δ*IP*_1,_*_Z_*. Now instead of just *Z*, we need to recognize a separate *Z*_P_ for proton count and *Z*_e_ for electron count, and instead of just *M*, we need to be clear that we mean *M*(*Z*_P_).

For single electron removal, we generally need the scaling of *IP*_1,1_ as:

(11) $${IP}_{1,1}Z/M\to {IP}_{1,1}\sqrt{Z_{\text{p}}Z_{\text{e}}}/M(Z_{\text{p}})$$

For the destruction of the original electron cluster, we need scaling of Δ*IP*_1,_*_Z_* as:

(12) $$\Delta {IP}_{1,Z}Z/M\to \Delta {IP}_{1,Z_{\text{e}}}Z_{\text{e}}/M(Z_{\text{p}})$$

along with an energy associated with the creation of the resultant electron cluster:

(13) $$-\Delta {IP}_{1,(Z_{\text{e}}-1)}(Z_{\text{e}}-1)/M(Z_{\text{p}})$$

This last energy increment will be cancelled if the electron removal process continues. Otherwise, depending on sign, it can represent heat lost to the environment, or taken from the environment.

For single electron addition, we need similar terms with some signs reversed. First, the baseline energy for adding (opposite of removing) an electron:

(14) $$-{IP}_{1,1}Z/M\to -{IP}_{1,1}\sqrt{Z_{\text{p}}Z_{\text{e}}}/M(Z_{\text{p}})$$

with the energy for destruction of the original electron cluster

(15) $$\Delta {IP}_{1,Z}Z/M\to \Delta {IP}_{1,Z_{\text{e}}}Z_{\text{e}}/M(Z_{p})$$

and the energy for the creation of the resultant electron cluster:

(16) $$-\Delta {IP}_{1,(Z_{\text{e}}+1)}(Z_{\text{e}}+1)/M(Z_{\text{p}})$$

The use of these formulae is illustrated with the analyses in the next Sections.

## 3. Ordinary Water

Ordinary water is made of two ordinary Hydrogen atoms and an ordinary Oxygen atom. To conduct an analysis of it, we need basic information about Hydrogen and Oxygen.

The development of the relevant information about **Hydrogen** goes as follows:

**Write Formulae:**

H1→H1+:IP1,1×1/M1H1→H1-:-IP1,1×1×2/M1-ΔIP1,2×2/M1

**Insert Data (eV):**

H1→H1+:14.250×1/1.008 eVH1→H1-:-14.250×1.4142/1.008-35.625×2/1.008 eV

**Evaluate Formulae:**

H1→H1+:14.1369 eVH1→H1-:-19.9924-70.6845=-90.6769 eV

The development of the relevant information about **Oxygen** goes as follows:

**Write Formulae:**

O8→O8+:IP1,1×8/M8+ΔIP1,8×8/M8-ΔIP1,7×7/M8O8+→O8++:IP1,1×8×7/M8+ΔIP1,7×7/M8-ΔIP1,6×6/M8O8→O8-:-IP1,1×8×9/M8-ΔIP1,9×9/M8+ΔIP1,8×8/M8O8-→O8--:-IP1,1×8×10/M8-ΔIP1,10×10/M8+ΔIP1,9×9/M8

**Insert Data (eV):**

O8→O8+:14.250×8/15.999+13.031×8/15.999-13.031×7/15.999 eVO8+→O8++:14.250×7.4833/15.999+13.031×7/15.999-7.320×6/15.999 eVO8→O8-:-14.250×8.4853/15.999-20.254×9/15.999+13.031×8/15.999 eVO8-→O8--:-14.250×8.9443/15.999-29.391×10/15.999+20.254×9/15.999 eV

**Evaluate Formulae:**

O8→O8+:7.1254+6.5159-5.7014=7.9399 eVO8+→O8++:6.6652+5.7014-2.7452=9.6214 eVO8→O8-:-7.5577-11.3936+6.5159=-12.4354 eVO8-→O8--:-7.9665-18.3705+11.3936=-14.9434 eV

**Summarize for Convenience:**

O8→O8++:7.9399+9.6214=17.5613 eVO8→O8--:-12.4354-14.9434=-27.3788 eV

Water is known to dissociate into the naked proton H^+^ and the hydroxyl radical OH^−^. The hydroxyl radical has to be the combination of ions O^− −^ + H^+^, the formation of which takes −27.3788 + 14.1369 = −13.2419 eV; there is not an alternative form using H^−^, because then O would have to be neutral.

So it is natural to consider H_2_O as H^+^ + OH^−^, or equivalently 2H^+^+ O^− −^. Forming water as H^+^ + OH^−^, or equivalently 2H^+^+ O^− −^, takes 14.1369 − 13.2419 = + 0.8950 eV, or equivalently 2 × 14.1369 − 27.3788 = +0.8950 eV, a slightly positive energy.

A molecule with a positive energy is not completely stable. It will tend to dissociate into the ions H^+^ and OH^−^, as ordinary water in fact does. But ordinary water does not dissociate very much. Only about one part in 10^7^ will be dissociated at any given moment. So water as 2H^+^+ O^− −^ isn’t the complete story.

I have previously considered [5] an additional feature that is analogous to pair formation between electrons, known in Physics as Cooper pairing, and very evident in Chemistry in the first ionization potential of Helium. The posited additional feature is pair formation between two H^+^’s; *i.e*. two naked protons.

But here I want to consider another possibility first. A plausible ionic configuration for water is 2H^−^+ O^++^. The transition ^1^H → H^−^ takes −19.9924 − 70.6845 + 0 = −90.6769 eV, and the transition _8_O → _8_O^++^ takes 17.5613 eV. So making H_2_O as 2H^−^ and O^++^ takes 2 × (−90.6769) + 17.5613 = −163.7925 eV. This is much more energetically favorable than making H_2_O as 2H^+^+ O^− −^, at +0.8950 eV. So 2H^−^+ O^++^ is a better candidate for the ionic configuration of ordinary water.

This ionic configuration helps explain why the normal water molecule is bent to an angle that recalls a tetrahedron? Indeed it does help. Imagine the heavy O^++^ ion with four satellites around it: two H nuclei (protons), and the two 2e subsystems (electron pairs). The two protons go to two corners of the tetrahedron, and the two electron pairs go to the other two corners.

And how about the polarization of ordinary water molecules? Does the ionic configuration explain that? Of course it does: The typical tetrahedral water molecule with two negative corners and two positive corners is, of course, polarized.

## 4. Physical States of Water

The polarization of ordinary water helps one water molecule attract another, and gradually form into a solid crystal: ice. There is an interesting feature about ordinary water ice: unlike just about anything else, water expands upon freezing. Does the vision of normal water as 2H^−^+ O^++^ in a tetrahedral arrangement help explain that odd property? Indeed it does help. The electron pairs have to orbit their respective protons, and being identical, they orbit in synchrony. That makes the whole tetrahedron spin about an axis that is the tetrahedron edge connecting the two protons. A tetrahedron spinning on an axis that is an edge is a lop-sided occupier of space. It sweeps out a volume that is larger than that of the tetrahedron, shaped like two regular cones joined at their circular bases, and slightly truncated at their apexes. That double-cone sweep volume is the minimum volume that a water molecule can occupy.

When the water is liquid, the spinning tetrahedrons can tolerate other spinning tetrahedrons temporarily invading any temporarily vacant space: they can bounce, or otherwise adjust. But when water is frozen, adjustments are not possible. Each spinning tetrahedron needs to have sole ownership of the volume of space in which it spins. So upon freezing, water expands.

We could really think about the freezing of water the other way around: upon melting, water contracts, because the flexibility of the liquid state permits ‘timesharing’ of the physical ‘real estate’.

And what of snowflakes? Why do they have their obvious hexagonal symmetry? The double-cone image helps with this question too. Obviously, the most efficient packing arrangement on a plane is one double cone surrounded by six others; *i.e*., hexagonal packing.

Snowflakes originate with nanostructures of seven molecules. Those nanostructures are perhaps the first ever to produce wonder in the human mind! Beyond this lovely thought, water is a pretty good example with which to talk about the physical states of matter generally.

This Section investigates the relationships between the macro states of matter—solid, liquid, gas, and plasma—and the micro states of ionization—neutral, singly ionized, doubly ionized, and so on. Readily available data show that boiling points and melting points follow a pattern, related to the pattern that first-order ionization potentials follow. Such patterning suggests that observed boiling and melting are related to hidden changes in ion populations. That would mean that the macroscopic states of matter are related to the microscopic states of atoms. This chapter poses a hypothesis about the relationship, and investigates the hypothesis in a quantitative way. The story involves the Planck energy distribution for black body radiation as a background for transitions between ionization states.

It is a source for photons of appropriate energy to provoke transitions, and it is a dumping ground for waste heat from spontaneous transitions. The state changes themselves often have a cascade character: the background supports the first change, and then some subsequent changes occur spontaneously. That scenario can make macroscopic state changes look as abrupt as they do.

This story goes far back to antiquity. Aristotle identified the macroscopic states of matter as Earth, Air, Fire, and Water. Seen in retrospect, that is an amazingly good categorization. The modern view has those same four macro states, but reordered as Earth, Water, Air, and Fire, and renamed as Solid, Liquid, Gas, and Plasma.

But now there are also many sub-categories that are specifically acknowledged. A Solid can be Conductor, a Super Conductor, a Semi Conductor, an Insulator, *etc*. A Liquid can be a Fluid, a Super Fluid, a Solvent, an Oil, a Plastic, a Pyroclastic, a Glass, a Conductor or an Insulator, a Solution, a Suspension, *etc*. A Gas can be Inert, Explosive, and so on, A Plasma can be Hot, Cold, *etc*.

### 4.1. Macroscopic Physical States and Microscopic Ionization States

It is reasonable to consider the possibility that macroscopic states of matter have some correlation with the microscopic ionization states, especially since one of the four main macroscopic states, namely the plasma state, has absolutely *everything* to do with ionization states.

What is the key similarity between macroscopic states of matter and ionization states? It is energy.

In the case of the four main macroscopic states of matter, state transitions can be correlated with changes in temperature and pressure, which relate to thermal and mechanical energy.

As for the various sub-categories within the four main macroscopic states, those too must have something to do with energy, but more in the nature of chemical-energy change, or configuration-energy change.

It appears that generally the difference between one macroscopic state of matter and another is some pressure × volume-change energy and/or some temperature × entropy-change energy.

In the case of ionization states, the difference between one state and another is electromagnetic energy, and, possibly, some thermal energy. To accomplish a change of ionization state, electromagnetic work may be invested, and heat may be dumped. The final energy of an ionization state is the cumulative sum of work increments invested and heat increments dumped in getting from the neutral state to the ionized state.

This Section is limited to just the four macroscopic states of matter: solid, liquid, gas, and plasma. Data about these macroscopic state changes are usually provided as point values of temperatures: melting points and boiling points. (As for excitation to the plasma state, that seems more difficult to document with temperature data.)

**Figure 3:**
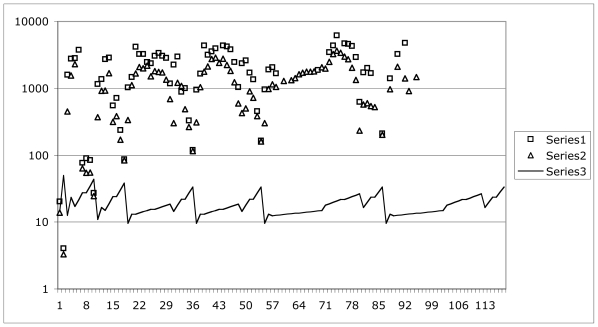
Correlation between state-change temperatures and ionization potentials.

Figure 3 shows reported melting points and boiling points, in comparison to first-order ionization potentials. The horizontal axis is the nuclear charge *Z* of the elements. The vertical axis is electron volts for ionization potentials, and degrees Kelvin for boiling points (series 1, squares) and melting points (series 2, triangles). The vertical scale is logarithmic to accommodate a large dynamic range. The solid line (series 3) reproduces the first order *IP*’s, or *M/Z*-scaled ionization potentials from Figure 1.

Observe that the dynamic range of boiling and melting temperatures is huge compared to the dynamic range of *IP*’s: we need four decades for temperatures, *vs*. one decade for *IP*’s. The temperature range for the liquid state varies from nearly a factor of 5.5 (*i.e*., very substantial range for liquid state) down to nearly a factor of 1 (*i.e*., no range at all for liquid state). There is a clue here that the liquid state is rare, so we are lucky that our planet, at least, has plenty of liquid water for us.

Despite the huge difference in dynamic range, the temperature data are clearly correlated with the *IP* data. The correlations are rough, but very compelling. What is actually going on here? To investigate this question, we require a hypothesis to test. My candidate hypothesis is that:

- The solid state involves ions and/or radicals existing in pairs that are in negative energy states;
- The liquid state involves neutral atoms and/or molecules, along with some ions existing in pairs that are mostly in negative energy states;
- The gas state involves neutral atoms and/or molecules, along with some ions existing in pairs that are mostly in positive energy states;
- The plasma state is composed significantly of ions existing in pairs that are in positive energy states.

### 4.2. Physical States and Ionization States

**Figure 4:**
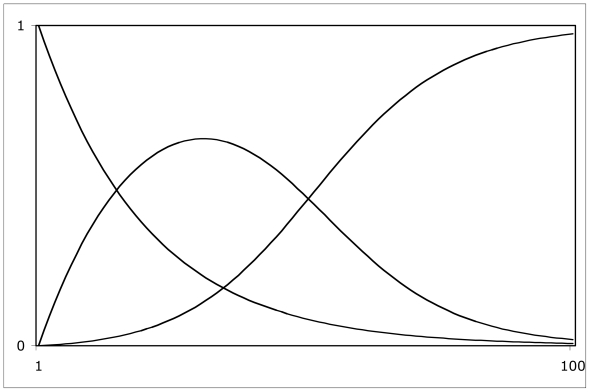
Population fractions of three regimes of ionization state energy.

Figure 4 offers a conceptual structure for the problem. The horizontal axis represents temperature, ranging from absolute zero to some very high temperature, represented by the ‘1’ at the right end. The vertical axis represents the population fraction of atom pairs in different ionization states. The energy of an ionized state is the cumulative result of work increments required and heat increments dumped in getting from the neutral state to the ionized state. It can be negative, positive, or zero. The three curves represent these three regimes of ionization state energy. The left curve, consistently descending with temperature, represents the fraction of ion pairs that are to be found in negative energy states. The right curve, consistently increasing with temperature, represents the fraction of ion pairs that are to be found in positive energy states. The middle curve, first increasing with temperature and then decreasing with temperature, represents the fraction of atom pairs to be found in the neutral, un-ionized state.

Figure 4 is quite generic. Its particular realization for a particular substance may be shifted right or left, or have the middle crossing point higher or lower, with the middle bump correspondingly lower or higher. *Much* higher is possible; you know this is true if you think about water: the proportion of H^+^ is only about 1 × 10^7^.

The three curves exhibit three crossing points, and they thereby define four temperature ranges. This situation invites consideration of the possibility that these four temperature ranges correlate with the four top-level states of matter: solid, liquid, gas, and plasma.

But temperature *T* is not the only independent variable. There is always at least one other. Pressure *P* can be taken as the other one. So macroscopic state changes are not adequately characterized as a point over a one-dimensional temperature axis; they need a line on a two-dimensional *P*, *T* phase diagram.

**Figure 5:**
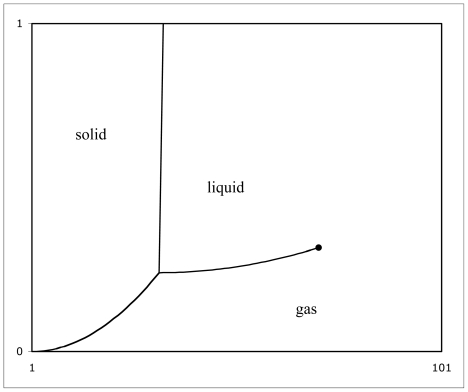
A generic phase diagram.

Figure 5 shows what a generic phase diagram looks like. The horizontal axis represents temperature *T*, and the vertical axis represents pressure *P*. The 1 on the horizontal axis corresponds to absolute zero temperature. The 101 means the plot is constructed from 101 temperature data points. The 0 on the vertical axis means zero pressure, and the 1 means the maximum pressure plotted, whatever that might actually be.

The left side of the picture corresponds to low temperature, and hence the solid state. The middle part of the picture represents higher temperature and hence the liquid state. The bottom of the picture represents low pressure, and hence the gas state.

The short curved line segment in the lower left marks transitions directly between solid and gas states, called ‘sublimation’ in the direction of solid-to-gas. Sublimation is an example of a situation wherein not all four states of matter occur. At low pressure, the liquid state does not occur; the solid state goes directly to the gas state.

The nearly vertical line marks the more usual transitions between solid and liquid. Observe that over most of the pressure range, these transitions occur at nearly the same temperature. So that is why the idea of ‘melting point’, or ‘freezing point’, is a pretty reliable one to put a single number to. However, pressure does affect melting temperature a little bit. You know that this is true, if you ever go ice-skating. The second curved line segment marks transitions between liquid and gas. It depends noticeably on pressure. You know that this is true, if you ever go camping at various mountain altitudes. That makes the idea of ‘boiling point’ or ‘condensation point’ tough to put a single number to.

The point on Figure 5 where the three lines meet is called the ‘triple point’. Three states of matter, solid, liquid, and gas, can co-exist there.

The point on Figure 5 marked by a big black dot is called the ‘critical point’. The distinction between liquid and gas simply disappears there. Matter in this condition simply goes ‘opalescent’. This is a big mystery, worthy of much research. It begs the questions: what about points not beyond, but beside the critical point? Where exactly does the distinction between liquid and gas reappear?

**Figure 6:**
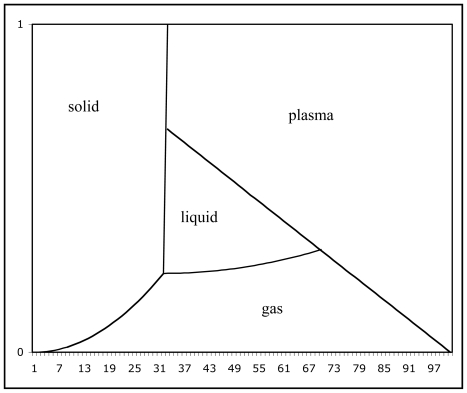
A phase diagram with the plasma state included.

Figure 5 overall begs an even bigger question: what about the plasma state? Unfortunately, it hasn’t been customary in the past to include the plasma state on phase diagrams. So including it constitutes an open research topic. Here we can only offer a few remarks. Consider Figure 6.

If the plasma state were included on phase diagrams, where might the transitions to plasma state go? I suspect a nexus to the critical point. I don’t really believe Nature does a ‘termination’ at the critical point; it more likely does another triple point, this one involving liquid, gas, and plasma. If so, where would the transition lines for liquid/plasma and gas/plasma likely go? It seems clear that for temperatures high enough, the plasma state trumps the gas state, and for pressures high enough, the solid state trumps the plasma state. So I expect the lines missing from Figure 5 to go generally diagonal, downward to the right, as illustrated by the new straight line shown on Figure 6. I do not mean to imply that the new lines should actually form this straight line; they could curve, they could change direction sharply at the critical point, or do whatever one can imagine; at present, we have no knowledge about such details. We only know that we should now go looking for them.

Observe that Figure 6 shows the liquid state limited to the totally surrounded, approximately triangular, area in the center. This means that, over the cosmological range of temperatures and pressures out there, the liquid state is something very rare. You know that this is true, if you are interested in space exploration, and follow NASA’s search for water.

Observe too that Figure 6 suggests yet another triple point, somewhere near the left top of the Figure. This third triple point involves the solid, liquid, and plasma states. It is my prediction that such a triple point exists, and will some day be observed.

Observe too that there are two more transitions of the sublimation type: a state of matter being skipped. Besides the transition from solid to gas, there is a transition from solid to plasma and a transition from liquid to plasma.

Can water illustrate for us any of these anticipated phenomena? It all depends on whether water can have anything like a plasma state. That question brings us to the next topic.

## 5. Isomers of Water

If water has anything like a plasma state, the molecule approaching that state is certainly not the ordinary tetrahedral water molecule. So we need to search out one or more isomers of water.

### 5.1. A Linear Isomer of Water

A linear isomer of water is thought to be the important constituent of the plasma-like phenomenon known as ‘Brown’s gas’. See, for example, [3,4]. Brown’s gas is of technological interest because it can perform welding and other technologically important tasks. One particularly interesting property of Brown’s gas is that it causes no harm when impinging on human flesh. Evidently, that is because the human flesh does not have metal content; flesh is essentially water, like the Brown’s gas itself is.

The ionic configuration 2H^+^+ O^− −^ previously displaced by 2H^−^+ O ^++^ as the candidate for ordinary tetrahedral water is actually quite interesting in this new context: it could be the ionic configuration for the linear isomer of water in Brown’s gas. Because all of the electrons in 2H^+^+ O^− −^ are in the possession of the Oxygen nucleus, the two naked Hydrogen nuclei can settle along the poles of the Oxygen system. So unlike the 2H^−^+ O ^++^ isomer, the 2H^+^+ O^− −^ isomer is not polarized.

Section 3 determined that the formation of water in the ionic configuration 2H^+^+ O^− −^ takes 14.1369 − 13.2419 = + 0.8950 eV, or equivalently 2 × 14.1369 − 27.3788 = + 0.8950 eV. This is a slightly positive energy. By contrast, the formation of water in the ionic configuration 2H^−^+ O ^++^ takes 2 × (−90.6769) + 17.5613 = −163.7925 eV. This is a very negative energy.

Section 4 posited an association between the energy of ionic configuration and physical state of matter. Observe that Brown’s gas fits the posited association: under conditions where ordinary tetrahedral water is liquid, the linear isomer is a gas. Observe too that, in an application like welding, Brown’s gas can be described as ‘burning’. But it doesn’t burn in the usual sense: by oxidation. It need not consume any oxygen; it can just revert to its normal water isomer. The release of energy makes a release of light, and at that moment the Brown’s gas may reasonably be regarded as being in the plasma state.

The whole situation with Brown’s gas can be compared to that of methane CH^4^. I previously analyzed the ionic configuration C^4−^ + 4H^+^ [5]. The relevant information about Carbon is developed as follows:

**Write Formulae:**

C6→C6-:-IP1,1×6×7/M6-ΔIP1,7×7/M6+ΔIP1,6×6/M6C6-→C6--:-IP1,1×6×8/M6-ΔIP1,8×8/M6+ΔIP1,7×7/M6C6--→C63-:-IP1,1×6×9/M6-ΔIP1,9×9/M6+ΔIP1,8×8/M6C63-→C64-:-IP1,1×6×10/M6-ΔIP1,10×10/M6+ΔIP1,9×9/M6

**Insert Data (eV):**

C6→C6-:-14.250×6.4807/12.011-13.031×7/12.011+7.320×6/12.011 eVC6-→C6--:-14.250×6.9282/12.011-13.031×8/12.011+13.031×7/12.011 eVC6--→C63-:-14.250×7.3485/12.011-20.254×9/12.011+13.031×8/12.011 eVC63-→C64-:-14.250×7.7460/12.011-29.391×10/12.011+20.254×9/12.011 eV

**Evaluate Formulae:**

C6→C6-:-7.688-7.5945+3.6566=-11.6267 eVC6-→C6--:-8.2197-8.6794+7.5945=-9.3046 eVC6--→C63-:-8.7184-15.1766+8.6794=-15.2156 eVC63-→C64-:-9.1900-24.4701+15.1766=-18.4835 eV

**Summarize for Convenience:**

C6→C64-:-11.6267-9.3046-15.2156-18.4835=-54.6304 eV

About **Hydrogen**, we already know _1_H → _1_H^+^: 14.1369 eV, so we have

$$4_{1}\text{H}\to 4_{1}{\text{H}}^{+}:4\times 14.1369=56.5476\;\text{eV}.$$

So forming methane as C^4−^ + 4H^+^ takes −54.6304 + 56.5476 = +1.9172 eV. This is just over twice the +0.8950 eV that it takes to make water as 2H^+^ + O^−−^. So both of these molecules in these ionic configurations are respectable fuels. The difference is that to release energy, methane has to burn oxygen, whereas linear water does not; it only has to revert to its tetrahedral isomer.

It is interesting that, like water, methane too has an alternative ionic configuration with charge signs reversed: C^4+^ + 4H^−^. To analyze this, the relevant information about Carbon is developed as

**Write Formulae:**

C6→C6+:IP1,1×6/M6+ΔIP1,6×6/M6-ΔIP1,5×5/M6C6+→C6++:IP1,1×6×5/M6+ΔIP1,5×5/M6-ΔIP1,4×4/M6C6++→C63+:IP1,1×6×4/M6+ΔIP1,4×4/M6-ΔIP1,3×3/M6C63+→C64+:IP1,1×6×3/M6+ΔIP1,3×3/M6-ΔIP1,2×2/M16

**Insert Data (eV):**

C6→C6+:14.250×6/12.011+7.320×6/12.011-2.805×5/12.011 eVC6+→C6++:14.250×5.4772/12.011+2.805×5/12.011-9.077×4/12.011 eVC6++→C63+:14.250×4.8990/12.011+9.077×4/12.011-(-1.781)×3/12.011 eVC63+→C64+:14.250×4.2426/12.011+(-1.781)×3/12.011-35.625×2/12.011 eV

**Evaluate Formulae:**

C6→C6+:7.1185+3.6566-1.1677=9.6074 eVC6+→C6++:6.4982+1.1678-3.0229=4.6431 eVC6++→C63+:5.8122+3.0229+0.4448=9.2799 eVC63+→C64+:5.0335-0.4448-5.9321=-1.3434 eV

**Summarize for Convenience:**

C6→C64+:9.6074+4.6431+9.2799-1.3434=22.1870 eV

About **Hydrogen**, we already know _1_H → _1_H^+^: −19.9924 − 70.6845 = −90.6769 eV

So we have

4H1→4H1-:4×(-90.6769)=-362.7076 eV

So forming methane as C^4+^ + 4H^−^ takes 22.1870 − 362.7076 = −340.5206 eV.

This methane molecule appears to be extremely stable energetically. But it might not be so stable geometrically, because H^−^ is very large compared to H^+^. So forming methane as C^4+^ + 4H^−^ might require high pressure.

One place that automatically provides high pressure is the deep ocean. So C^4+^ + 4H^−^ looks like a good candidate configuration to expect within the vast deposits of methane known to exist at the bottom of the world’s oceans.

Both of the two methane isomers mentioned above, C^4−^ + 4H^+^ and C^4+^ + 4H^−^, are tetrahedral in shape, and are not polarized.

### 5.2. Isomers of Water Involving Proton Pairs

So far, water and methane have a lot in common: each has one fairly inert isomer, and one fuel-ready isomer. Let us now consider one more potential variation on the molecules involving H^+^’s, *i.e*., naked protons. Suppose that naked protons can do what electrons most like to do: form binary pairs. Let such pairs be represented the notation (2H^+^).

Applied to the C^4−^ + 4H^+^ fuel-ready isomer of methane CH_4_, such a process could produce another isomer. Let this new isomer be represented by the formulaic notation C^4−^ + 2(2H^+^) showing that it involves two protons pairs. Let it be represented by the pictorial layout (2H^+^) : C^4−^ : (2H^+^), showing that it is an un-polarized linear molecule with two strongly positive ends. This isomer of methane could exist, but it’s potential utility is unclear.

But the pairing process could also produce yet another isomer, with only one proton pair. The formulaic notation would be C^4−^ + 2H^+^ + (2H^+^), and the pictorial representation would be $\begin{array}{c} {\text{H}}^{+}\cdot \\ {\text{H}}^{+}\cdot \end{array}{\text{C}}^{4-}\cdot (2{\text{H}}^{+})$. (The little dots mean ‘chemical bonds’, a mysterious concept worthy of a whole book on its own.) This molecule is not symmetric, and so it is polarized. Its polarization can trigger a cascade process that can lead to heavier and heavier hydrocarbons. That is, polarized methane CH_4_ can lead to ethane C_2_H_6_, propane C_3_H_8_, butane C_4_H_10_, and so on.

In more detail, the ethane C_2_H_6_ molecule with a proton pair would have formulaic representation 2C^3−^ + 4H^+^ + (2H^+^), and pictorial representation

$$\begin{array}{c} {\text{H}}^{+}\cdot \\ {\text{H}}^{+}\cdot \end{array}{\text{C}}^{3-}\cdot (2{\text{H}}^{+})\cdot {\text{C}}^{3-}\begin{array}{c} \cdot {\text{H}}^{+}\\ \cdot {\text{H}}^{+} \end{array}$$

The propane C_3_H_8_ molecule with proton pairs would have formulaic representation 3C^3−^ + 6H^+^ + 2(2H^+^) and pictorial representation

$$\begin{array}{c} {\text{H}}^{+}\cdot \\ {\text{H}}^{+}\cdot \end{array}{\text{C}}^{3-}\cdot (2{\text{H}}^{+})\cdot {\text{C}}^{3-}\cdot (2{\text{H}}^{+})\cdot {\text{C}}^{3-}\begin{array}{c} \cdot {\text{H}}^{+}\\ \cdot {\text{H}}^{+} \end{array}$$

The butane C_4_H_10_ molecule with proton pairs would have formulaic representation 4C^3−^ + 4H^+^ + 3(2H^+^)and pictorial representation

$$\begin{array}{c} {\text{H}}^{+}\cdot \\ {\text{H}}^{+}\cdot \end{array}{\text{C}}^{3-}\cdot (2{\text{H}}^{+})\cdot {\text{C}}^{3-}\cdot (2{\text{H}}^{+})\cdot {\text{C}}^{3-}\cdot (2{\text{H}}^{+})\cdot {\text{C}}^{3-}\begin{array}{c} \cdot {\text{H}}^{+}\\ \cdot {\text{H}}^{+} \end{array}$$

What is happening at each step here is that a polarized methane unit $\begin{array}{c} {\text{H}}^{+}\cdot \\ {\text{H}}^{+}\cdot \end{array}{\text{C}}^{4-}:(2{\text{H}}^{+})$ with its proton pair meets the growing molecule, reacts with it in a way such that one hydrogen gas molecule gets expelled, and the next longer molecule gets formed. This process is a kind of ‘polymerization’, although not the usual kind, with addition onto the end of the molecule; instead, it features insertion somewhere into the middle of the growing molecule. (The vision that heavy hydrocarbons are created from light hydrocarbons under extreme temperature / pressure conditions comes from [6])

All of the chain hydrocarbon molecules can be thought of as polymer molecules, and of course polymer molecules can be thought of as nanostructures. So, is there, as there was so often above, some analogous situation and ensuing process with water? Consider the linear isomer of water, 2H^+^+ O^− −^. The proton pairing process would produce another isomer. Its formulaic representation would be O^− −^ + (2H^+^) and its pictorial representation would be O^− −^ (2H^+^). This is a doubly polarized, linear molecule that is more compact than 2H^+^+ O^− −^ is. The polarization and the linear shape would encourage such water to form linear polymeric structures. But the situation is very different from that with methane. The water polymerization process just features molecular alignment. The first polymer formed just has formulaic representation 2O^− −^ + 2(2H^+^) and visual representation O^− −^ (2H^+^) O^− −^ (2H^+^). There is no chemical reaction to cement that alignment once it is achieved. That is, there is no analog to the expulsion of the Hydrogen molecule that occurs in the creation of the hydrocarbon polymers. So there is nothing to keep the aligned water molecules from reverting to their un-aligned condition.

Back in the late 1960’s and early 1970’s, there was a lot of controversy in the chemistry literature and the popular press over the purported existence of so-called ‘polywater’. The name refers to water that was put through a physical gauntlet involving passage through narrow quartz capillary tubes. The physical properties of the resulting ‘polywater’ were described as rather polymer-like. But the claimed phenomenon of polywater could not be reliably reproduced at the time, despite much investment and effort, and was eventually dismissed as an unreal phantom. The whole episode is today regarded as an example of ‘pathological science’.

Now we can see why nothing came of polywater. Polymerization of water is possible, yes, but it has absolutely no staying power whatsoever. One simply cannot create samples of polywater, package them, and carry them about to independent testing laboratories—as was attempted way back then.

Polywater is, however, worth remembering, and studying more rationally, since the polymerization idea involved in it has its analog in the important area of hydrocarbon fuels.

Presumably, the corresponding water process has to begin with the ordinary tetrahedral isomer of water, 2H^−^+ O ^++^. Then the 2H^+^+ O^− −^ linear isomer of water has to be created. The process for making this linear isomer is not fully revealed in the literature. But perhaps AC can offer some reasonable guesses about it. Variables that can be controlled include temperature and pressure and electrical current. Electrical current appears to be the most important one.

Then the proton-pairing process has to be triggered. This is probably not so difficult, considering how easy, even unavoidable, it is in the case of electron pairing.

Then the polarized molecules have to be brought into close proximity. The combination of low temperature and high pressure would help put molecules into the requisite close proximity.

Then the polymerization has to be sensed immediately, *in situ*. Optical methods involving polarized light could be useful for this.

## 6. Water Containing Isotopes of Its Constituent Atoms

Both the Hydrogen and the Oxygen in water can occur in isotopes, with different neutron counts in the nucleus. The technologically important isotopes are those of Hydrogen. There are three of them: normal Hydrogen (with only the one proton), Deuterium (one proton with one neutron) and Tritium (one proton with two neutrons).

The heavy isotopes of Hydrogen are found in all water, but in greater concentration in so called ‘heavy water’. Heavy water can be produced by evaporation of ordinary seawater, because the lighter water molecules will evaporate before the heavier water molecules.

Heavy water is a raw material for all sorts of fusion experiments. Fusion experimenters typically get heavy water from special-purpose vendors. The experimenters may not fully understand how hard heavy water is to make, or exactly what the vendor has actually provided. For example, suppose the production process starts with seawater, and uses boiling to remove normal H_2_O, and/or centrifuging to increase the concentration of heavier species, which include DHO and D_2_O and THO and TDO and T_2_O. In what proportions do these heavier species occur? Who knows! But my guess is that DHO is the dominant heavy-water species available, not D_2_O (and certainly not anything involving T), and that significant ordinary H_2_O is still left in any heavy-water sample. But fortunately, that doesn’t matter, so long as at least some heavy nuclei are present.

What matters more is to find a way to meet the fundamental requirements for fusion to occur. These include:

1. Removal of electrons from the immediate environs of nuclei to be fused;
2. Forcing of the naked nuclei into proximity sufficient for their attraction by nuclear forces to dominate their natural Coulomb repulsion.

The traditional Hot Fusion (HF) does these jobs in a brute-force way. To meet Requirement 1, the oxygen is separated out, leaving gas molecules H_2_, HD, D_2_, *etc*., which are heated into its plasma state. To meet Requirement 2, the plasma is confined and compressed with a huge magnetic field.

All of that is exceedingly difficult, and difficulty creates motivation to seek alternatives using less brute force and more subtlety and guile. In recent decades, many efforts to develop alternatives to HF have been characterized by the title ‘Cold Fusion’ (CF).

Arising from empiricism rather than from theory, CF has been much maligned. But Algebraic Chemistry (AC) offers at least a quantitative modeling tool with which to approach CF objectively. The following Sub-Sections develop this story.

### 6.1. Data on Isotopes of Hydrogen

The development of the relevant information about all the individual Hydrogen isotopes follows. Notice that the masses of the individual isotopes are integers, as opposed to the real-number average value seen earlier in the paper.

**For normal Hydrogen**:

**Write Formulae:**

H1→H1+:IP1,1×1/1;H1→H1-:-IP1,1×1×2/1-ΔIP1,2×2/1

**Insert Data (eV):**

H1→H1+:14.250×1/1 eV;H1→H1-:-14.250×1.4142/1-35.625×2/1 eV

**Insert Data (eV):**

H1→H1+:14.250×1/1 eV;H1→H1-:-14.250×1/1-35.625×2/1 eV

**Evaluate Formulae:**

H1→H1+:14.250 eV;H1→H1-:-20.1525-71.2500+0=-91.4025 eV

**For Deuterium:**

**Write Formulae:**

D1→D1+:IP1,1×1/2 eV; D1→D1-:-IP1,1×1×2/2-ΔIP1,2×2/2

**Insert Data (eV):**

D1→D1+:14.250×1/2 eV; D1→D1-:-14.250×1.4142/2-35.625×2/2 eV

**Evaluate Formulae:**

D1→D1+:7.125 eV;D1→D1-:-10.07625-35.625=-45.70125 eV

**For Tritium:**

**Write Formulae:**

T1→T1+:IP1,1×1/3 eV; T1→T1-:-IP1,1×1×2/3-ΔIP1,2×2/3

**Insert Data (eV):**

T1→T1+:14.250×1/3 eV; T1→T1-:-14.250×1.4142/3-35.625×2/3 eV

**Evaluate Formulae:**

T1→T1+:4.750 eV; T1→T1-:-6.71745-23.750+0=-30.46745 eV

### 6.2. Comments on Cold Fusion

Here I wish to comment on some beliefs about cold fusion that are commonly articulated, and might be wrong, and some other ideas that haven’t been articulated before, and may deserve some study.

Those who are skeptical about CF have often pointed to its meager generation of neutrons, which are common byproducts of many of the more familiar nuclear fission processes. But there is a flaw in this sort of objection. **When we study the Periodic Table, we see that the proportion of neutrons in stable isotopes increases with atomic number.** That means fission reactions typically start with elements that have more neutrons than the daughter elements will need for stability. So the excess neutrons are liberated in the fission process. By contrast, fusion reactions do not occur between the abundant isotopes of input elements because those isotopes do not have enough neutrons to make a stable isotope of the product element. Fusion reactions need some heavy isotope(s) as input. And even then, the neutrons provided may be too valuable for any to be liberated. Observe that the chemical/nuclear reactions

(10) $$2\text{DHO}\to \text{He}+{\text{H}}_{2}+{\text{O}}_{2}\;\text{and }{\text{H}}_{2}\text{O}+\text{THO}\to \text{He}+{\text{H}}_{2}+{\text{O}}_{2}$$

can produce energy without producing any neutrons at all. Even though no other reactions seem able to do exactly that, do take note that:

**The fact that neutrons are absent from, or at low concentration in, the environs of a purported CF experiment does** **not** **mean that there is no fusion occurring.**

Those who are proponents of CF are obliged to justify the occurrence of any nuclear reaction at all in circumstances so modest as a CF cell provides: no high temperature, no high pressure, no magnetic confinement; in short, no big hardware.

A possible explanation is that the conditions of the CF cell produce a heavy version of Brown’s gas, including not only the linear isomer of H_2_O, but also linear isomers of DHO and THO, and possibly D_2_O and DTO and T_2_O too. The full set of linear isomers puts not only naked H’s, but also naked d ’s and naked T’s, in exposed positions, unshielded by electrons. Nuclear reactions are then possible: two D’s, or one T and one H, can fuse to make a normal ^4^He nucleus, or two D can make a T and an H, or a ^3^He and a neutron, *etc*. My guess is that the dominant nuclear reaction is 2D → ^4^He + γ, where γ is a gamma ray carrying the energy released. Other reactions can also produced some neutrons, which can then trigger other elements present to undergo fission. All these reactions amount to transmutations.

And what condition is it that the CF cell provides to make the linear isomers of water and heavy water? Basically, I believe it is an environment of chemical catalysis. The cathode in a CF cell is typically Palladium. That element comes from an area of the Periodic Table where famously catalytic metals are found. Some others are Silver, Platinum, and Gold. The CF anode is usually Platinum. Gold also sometimes appears, as a cathode. I have not seen Silver yet, but would not be surprised to see it. All these metals prefer not to be neutral, and upon encountering any other atom, will negotiate to give or take electrons. That is how they catalyze further reactions.

With Algebraic Chemistry, it is possible to calculate the energy costs and benefits of various electron rearrangements. So we can find out why a DHO molecule might get catalyzed into linear configuration in the CF cell.

The development of the relevant information about Palladium goes as follows:

**Write Formulae:**

P46d→P46d+:IP1,1×46/M46+ΔIP1,46×46/M46-ΔIP1,45×45/M46P46d+→P46d++:IP1,1×46×45/M46+ΔIP1,45×45/M46-ΔIP1,44×44/M46P46d→P46d-:-IP1,1×46×47/M46-ΔIP1,47×47/M46+ΔIP1,46×46/M46P46d-→P46d--:-IP1,1×46×48/M46-ΔIP1,48×48/M46+ΔIP1,47×47/M46

**Insert Data:**

P46d→P46d+:14.250×46/106.420+2.701×46/106.420-1.980×45/106.420 eVP46d+→P46d++:14.250×45.4973/106.420+1.980×45/106.420-1.289×44/106.420 eVP46d→P46d-:-14.250×46.4973/106.420-3.455×47/106.420+2.701×46/106.420 eVP46d-→P46d--:-14.250×46.9894/106.420-4.242×48/106.420+3.455×47/106.420 eV

**Evaluate Formulae:**

P46d→P46d+:6.1596+1.1675-0.8372=6.4899 eVP46d+→P46d++:6.0922+0.8372-0.5329=6.3964 eVP46d→P46d-:-6.2261-1.5259+1.1675=-6.5845 eVP46d-→P46d--:-6.2920-1.9133+1.5259=-6.6794 eV

**Summarize for Convenience:**

P46d→P46d++:6.4899+6.3964=12.8863 eVP46d→P46d--:-6.5845-6.6794=-13.2639 eV

In [7], Gordon talked about ‘co-deposition’, meaning co-introduction of Palladium and Deuterium into the CF cell, both in solution form. The added Palladium is in the form of the salt PdCl_2_, which presumably dissociates into the ions Pd^++^ and 2Cl^−^. The Deuterium is, I believe, in the form of DHO which, like normal water, does not dissociate very much, but stays as a molecule with ionic structure (D^−^ + H^−^ + O^+ +^). The available Pd^++^ ions can work on the normal DHO molecule to convert it to linear form, with ionic structure (D^+^ + H^+^ + O^− −^). There must be many pathways to this end result; indeed a ‘riotous profusion’ of possible pathways is typical of catalysis schemes. Here is just one reaction pathway, given for illustration of the principle involved:

$$({\text{D}}^{-}+{\text{H}}^{-}+{\text{O}}^{++})+10{\text{Pd}}^{++}+20{\text{e}}^{-}\to ({\text{D}}^{+}+{\text{H}}^{+}+{\text{O}}^{--})+10\text{Pd}$$

From the data provided above, this reaction takes

$$\begin{array}{c} 45.70125+91.4025-17.5613-10\times 12.8863+7.125+14.250-27.3788+10\times 0=45.70125\\ +91.4025-17.5613-128.863+7.125+14.250-27.3788=-15.32435\;\text{eV} \end{array}$$

Observe that the energy requirement for this reaction is negative, which means that this reaction will occur spontaneously.

Thus about the first requirement for fusion, removing electrons from subject nuclei, AC indicates that chemical catalysis can do the job. The catalysis converts normal DHO (analogous to normal water) into linear DHO (linear, like BG). The ionic structure of normal DHO keeps the D in association with two electrons, and so protected from intrusions to the nuclear level, but the ionic structure of linear DHO leaves the D exposed, unshielded by any electron.

With regard to the second requirement for fusion, confinement, CF does provide some of that in the form of Deuterium loading into the Palladium matrix of the cathode in the CF cell. But that is not enough confinement to cause neighbor-on-neighbor fusion inside the cathode [8]. Instead of fusion occurring interior to the cathode, fusion between the trapped cathode-loaded Deuterons and the exposed Deuterons in the electrolyte seems more likely. Basically, the Deuteron loading sets up the cathode like a dart board, and then the linear HDO molecules in the electrolyte, positively charged on the ends, are attracted to the cathode because it is negative, and they hit it like so many darts. When D ’s on those incident darts hit bull’s-eye D ’s on the target cathode, CF ensues.

## 7. Conclusions

This paper has shown that many physical behaviors of molecules and nanostructures are understandable in terms of ‘Algebraic Chemistry’. This name refers to a quantitative model for predicting the energy budget for all sorts of changes between different ionization states of atoms that are involved in chemical reactions and in changes of physical state. The paper states the formulae involved, and shows many examples of their use. The Appendix gives all the numerical data used here, plus similar data potentially useful for future studies involving additional elements.

The paper has suggested an association between macroscopic physical states of matter and microscopic ionization states of its constituent atoms. Some gaps in current knowledge are pointed out with the hope of stimulating future research.

About water in particular, the paper has argued that water does not ‘live’ in the ionization state to which it ‘dies’. If it did, it would dissociate more than it does. In detail, water lives with ionic configuration 2H^−^ + O^2+^, but it dies to H^+^ + OH^−^, which has overall ionic configuration 2H^+^ + O^− −^.

The paper has argued that water also has several interesting isomers corresponding to different ionization states. The 2H^+^ + O^− −^ ionization state is a linear isomer, apparently the key ingredient in socalled Brown’s gas, which is a fuel-like substance. The analysis developed here indicates that Brown’s gas works by a mechanism that is more akin to Spectroscopy than to Chemistry: it burns no Oxygen; it just relaxes to a less energetic ionic configuration.

An ionization state involving pairing of naked protons, analogous to the well-known Cooper pairing of electrons, is discussed. It could produce transient polymerization. So-called ‘polywater’ has a checkered past, and stands in need of rehabilitation. If it ever exists at all, it is totally ephemeral. But it may yet be detectable. It does not have an obvious practical utility, but the analogous process with hydrocarbons could be useful to know about.

The paper finally worked out some details for water with isotopes of Hydrogen in it. This information is useful for explaining what can be happening in ‘cold fusion’. This technology too has a difficult past. But it appears to have a potentially useful future.

## Figures and Tables

**Table 1:** First-order *IP*’s: fractional rises on sub-periods.

period	*L*	*N*	*l*	fraction	*l*	fraction	*l*	fraction	*l*	fraction
1	2	1	0	1						
2	8	2	0	1/2	1	3/4				
3	8	2	0	1/3	1	3/4				
4	18	3	0	1/4	2	5/18	1	2/3		
5	18	3	0	1/4	2	5/18	1	2/3		
6	32	4	0	1/4	3	7/48	2	5/16	1	9/16
7	32	4	0	1/4	3	7/48	2	5/16	1	9/16

## Appendix 1 Basic data on first-order ionization potentials

**Table:** Periods 1, 2 and 3.

Element	Charge *Z*	Mass *M*	Raw Data IP	Scaled Data *IP*	Model *IP*	Model Δ*IP*
H	1	1.008	13.610	13.718	14.250	0
He	2	4.003	24.606	49.244	49.875	35.625

Li	3	6.941	5.394	12.480	12.480	−1.781
Be	4	9.012	9.326	21.011	23.327	9.077
B	5	10.811	8.309	17.966	17.055	2.805
C	6	14.007	11.266	22.551	21.570	7.320
N	7	14.007	14.544	29.101	27.281	13.031
O	8	15.999	13.631	27.260	27.281	13.031
F	9	18.998	17.438	36.810	34.504	20.254
Ne	10	20.180	21.587	43.562	43.641	29.391

Na	11	22.990	5.145	10.753	10.910	−3.340
Mg	12	24.305	7.656	15.506	16.565	2.315
Al	13	26.982	5.996	12.444	14.923	0.673
Si	14	28.086	8.154	16.357	18.874	4.624
P	15	30.974	10.498	21.677	23.871	9.621
S	16	32.066	10.373	20.790	23.871	9.621
CI	17	35.453	12.977	27.063	30.192	15.942
Ar	18	39.948	15.778	35.017	38.186	23.936

Note above: *l* = 0 and *l* = 1 sub-periods are tied to the beginnings and ends of periods. Note following: *l* = 2 sub-periods float mid period, with their mid points tied to the overall period-rise.

**Table:** Periods 4 and 5.

Element	Charge *Z*	Mass *M*	Raw Data IP	Scaled Data *IP*	Model *IP*	Model Δ *IP*
K	19	39.098	4.346	8.944	9.546	−4.704
Ca	20	40.078	6.120	12.265	13.057	−1.193
Sc	21	44.956	6.546	14.013	13.057	−1.193
Ti	22	47.867	6.826	14.851	13.638	−0.612
V	23	50.942	6.743	14.934	14.244	−0.006
Cr	24	51.996	6.774	14.676	14.877	0.627
*Mn*	25	54.938	7.438	16.345	15.539	1.289
Fe	26	55.845	7.873	16.911	15.539	1.289
Co	27	58.933	7.863	17.163	16.229	1.980
Ni	28	58.693	7.645	16.026	16.951	2.701
Cu	29	63.546	7.728	16.934	17.705	3.455
Zn	30	65.390	9.398	20.485	18.492	4.242
Ga	31	69.723	6.006	13.509	14.494	0.244
Ge	32	72.610	7.905	17.936	17.860	3.610
As	33	74.922	9.824	22.303	22.007	7.757
Se	34	78.960	9.761	22.669	22.007	7.757
Br	35	79.904	11.826	26.998	27.116	12.866
Kr	36	83.800	14.015	32.623	33.412	19.162

Rb	37	85.468	4.180	9.657	9.546	−4.704
Sr	38	87.620	5.695	13.132	13.057	−1.193
Y	39	88.906	6.390	14.567	13.057	−1.193
Zr	40	91.224	6.846	15.614	13.638	−0.612
Nb	41	92.906	6.888	15.608	14.244	−0.006
Mo	42	95.940	7.106	16.232	14.877	0.627
Tc	43	98.000	7.282	16.597	15.539	1.289
Ru	44	101.070	7.376	16.942	15.539	1.289
Rh	45	102.906	7.469	17.080	16.230	1.980
Pd	46	106.420	8.351	19.319	16.951	2.701
Ag	47	107.868	7.583	17.403	17.705	3.455
Cd	48	112.411	9.004	21.087	18.492	4.242
In	49	114.818	5.788	13.563	14.494	0.244
Sn	50	118.710	7.355	17.462	17.860	3.610
Sb	51	121.760	8.651	20.655	22.007	7.757
Te	52	127.600	9.015	22.120	22.007	7.757
I	53	126.904	10.456	25.037	27.116	12.866
Xe	54	131.290	12.137	29.508	33.412	19.162

**Table:** Period 6.

Element	Charge *Z*	Mass *M*	Raw Data IP	Scaled Data *IP*	Model *IP*	Model Δ *IP*
Cs	55	132.905	3.900	9.425	9.546	−4.704
Ba	56	137.327	5.218	12.796	13.057	−1.192
La	57	138.906	5.581	13.600	12.393	−1.857
Ce	58	140.116	5.477	13.232	12.583	−1.667
Pr	59	140.908	5.425	12.957	12.776	−1.474
Nd	60	144.240	5.498	13.217	12.972	−1.278
Pm	61	145.000	5.550	13.192	13.171	−1.079
Sm	62	150.360	5.633	13.660	13.374	−0.876
Eu	63	151.964	5.674	13.687	13.579	−0.671
Gd	64	157.250	6.141	15.089	13.579	−0.671
Tb	65	158.925	5.851	14.305	13.787	−0.463
Dy	66	162.500	5.934	14.609	13.999	−0.251
Ho	67	164.930	6.027	14.836	14.213	−0.037
Er	68	167.260	6.110	15.029	14.431	0.181
Tm	69	168.934	6.183	15.137	14.653	0.403
Yb	70	170.040	6.255	15.463	14.878	0.627
Lu	71	174.967	5.436	13.395	17.860	3.610
Hf	72	178.490	7.054	17.487	18.755	4.505
Ta	73	180.948	7.894	19.568	19.696	5.446
W	74	183.840	7.988	19.844	20.684	6.434
Re	75	186.207	7.884	19.574	21.721	7.471
Os	76	190.230	8.714	21.811	21.721	7.471
Ir	77	192.217	9.129	22.788	22.811	8.560
Pt	78	195.076	9.025	22.571	23.955	9.705
Au	79	196.967	9.232	23.019	25.156	10.906
Hg	80	200.530	10.446	26.184	26.418	12.168
Tl	81	204.383	6.110	15.417	16.515	2.265
Pb	82	207.200	7.427	18.768	19.696	5.446
Bi	83	208.980	7.293	18.361	23.490	9.240
Po	84	209.000	8.423	20.958	23.490	9.240
At	85	210.000			28.015	13.765
Rn	86	222.000	10.757	27.769	33.412	19.164

Note: *l* = 3 (like *l* = 2) sub-periods float mid period, with their midpoints tied to the overall period-rise.

**Table:** Period 7.

Element	Charge *Z*	Mass *M*	Raw Data IP	Scaled Data *IP*	Model *IP*	Model *ΔIP*
Fr	87	223.000			9.546	−4.704
Ra	88	226.000	5.280	13.560	13.057	−1.193
Ac	89	227.000	6.950	17.727	12.393	−1.857
Th	90	232.038	6.089	15.699	12.583	−1.667
Pa	91	231.036	5.892	14.959	12.776	−1.474
U	92	238.029	6.203	16.050	12.972	−1.277
Np	93	237.000	6.276	15.994	13.171	−1.079
Pu	94	244.000	6.068	15.752	13.374	−0.876
Am	95	243.000	5.996	15.337	13.579	−0.671
Cm	96	247.000	6.027	15.507	13.579	−0.671
Bk	97	247.000	6.234	15.875	13.787	−0.463
Cf	98	251.000	6.307	16.154	13.999	−0.251
Es	99	252.000	6.421	16.345	14.213	−0.037
Fm	100	257.000	6.504	16.716	14.431	0.181
Md	101	258.000	6.587	16.827	14.653	0.403
No	102	259.000	6.660	16.911	14.877	0.627
Lf	103				17.859	3.610
Rf	104				18.755	4.505
Db	105				19.696	5.446
Sg	106				20.684	6.434
Bh	107				21.721	7.471
Hs	108				21.721	7.471
Mt	109				22.811	8.561
Ds	110				23.955	9.705
Rg	111				25.156	10.906
Cn	112				26.418	12.168
Uut	113				16.515	2.265
Uuq	114				17.019	2.769
Uup	115				23.490	9.240
Uuh	116				23.490	9.240
Uus	117				28.015	13.765
Uuo	118				33.412	19.162

## References

[1] Whitney C.K. (2009). Closing in on chemical bonds by opening up relativity theory. Int. J. Mol. Sci.

[2] Putz M.V. (2006). Systematic formulations for electronegativity and hardness and their atomic scales within density functional softness theory. Int. J. Quantum Chem.

[3] Eckman C. (2010). Plasma orbital expansion of the electrons in water. Proc. NPA.

[4] Santilli R.M. (2010). A new gaseous and combustible form of water. Int. J. Hydrogen Energy.

[5] Whitney C.K., Putz M.V. (2011). The Algebraic Chemistry of Molecules and Reactons. Quantum Frontiers of Atoms and Molecules.

[6] Scarborough A.A. (2008). Origins of Universal Systems.

[7] Gordon F. How Hot Is Cold Fusion?.

[8] Tadahiko M. (1998). Nuclear Transmutation: The Reality of Cold Fusion.

